# Weight control behaviors according to body weight status and accuracy of weight perceptions among Korean women: a nationwide population-based survey

**DOI:** 10.1038/s41598-019-45596-z

**Published:** 2019-06-24

**Authors:** Boyoung Park, Ha Na Cho, Eunji Choi, Da Hea Seo, Nam-Soon Kim, Eunja Park, Sue Kim, Yeong-Ran Park, Kui Son Choi, Yumie Rhee

**Affiliations:** 1 Department of Medicine, Hanyang University College of Medicine, Seoul, South Korea; 2 Department of Cancer Control and Population Health, Graduate School of Cancer Science and Policy, National Cancer Center, Goyang, South Korea; 3 Department of Endocrinology and Metabolism, School of Medicine, Inha University, Incheon, South Korea; 4 Health Care Policy Research Department, Korea Institute for Health and Social Affairs, Sejong, South Korea; 5 College of Nursing, Yonsei University, Seoul, South Korea; 6 Department of Silver Industry, Kangnam University, Yongin, South Korea; 7 Department of Internal Medicine, Endocrine Research Institute, Severance Hospital, Yonsei University College of Medicine, Seoul, South Korea

**Keywords:** Nutrition, Epidemiology

## Abstract

This study aimed to identify associations among self-perceived weight status, accuracy of weight perceptions, and weight control behaviors, including both healthy and unhealthy behaviors, in a large, nationally representative sample from an East Asian country. Data were collected from the 2016 Korean Study of Women’s Health Related Issues, a population-based, nationwide survey. Accurate weight perceptions were investigated by comparing body mass index (BMI) categories, based on self-reported height and weight, and weight perceptions. Weight control behaviors over the previous 12 months were additionally surveyed. Odds ratios (ORs) and 95% confidence intervals (CIs) are presented as an index of associations. Among normal weight, overweight, and obese women, 12.8%, 44.3%, and 17.4% under-assessed their weight; 17.9% of normal weight women over-assessed their weight. Both weight status according to BMI category and weight perceptions were strongly associated with having tried to lose weight. Exercise and diet (ate less) were the most commonly applied weight control behaviors. Misperception of weight was related to more unhealthy weight control behaviors and less healthy behaviors: Women who under-assessed their weight showed a lower tendency to engage in dieting (OR = 0.57, 95% CI = 0.43–0.75) and a greater tendency to fast/skip meals (OR = 1.47, 95% CI = 1.07–1.99). Meanwhile, normal weight or overweight women who over-assessed their weight were more likely to have engaged in fasting/skipping meals or using diet pills (OR = 5.72, 95% CI = 2.45–13.56 for fasting/skipping meal in overweight women; OR = 1.62, 95% CI = 1.15–2.29 and OR = 3.16, 95% CI = 1.15–8.23 for using diet pills in normal and overweight women). Inaccuracy of weight perceptions in any direction (over/under) were related to more unhealthy weight control behaviors and less healthy weight control behaviors, especially in normal and overweight women.

## Introduction

Having rapidly increased over the past few decades, the worldwide prevalences of obesity and overweight in 2013 were reportedly 36.9% in men and 38.0% in women^1^. The World Health Organization has labeled obesity as an important, but neglected, problem in public health^2^. Indeed, obesity has been shown to be associated with increased risks of various cardiovascular and metabolic comorbidities, as well as premature death^2^. While modest weight loss of 5–10% could improve risk factors for cardiovascular disease^3^, weight loss and maintaining weight loss are difficult to achieve^4^.

One’s perception of their body is an important facilitator of helping people to lose and maintain weight loss^5^. Moreover, it has been found to be vital to the success of weight control practices^6^. Self-body perceptions reflect an individual’s awareness of body weight status in relation to their actual body weight^7^. As an explanation of the mechanism linking self-body perception and weight control, it has been proposed that weight perceptions act as a starting point from which to attempt weight control and that attempts to change health behaviors are not likely to work in overweight or obese people lacking correct body perceptions^8,9^. Interestingly, several studies have shown that perceived weight status is more strongly correlated with weight loss attempts than measured weight status^10–12^. Negative effects of misperceived body weight status on weight control behaviors, however, have also been reported. People who perceived their weight status inaccurately were more likely to engage in extreme or unhealthy weight management practices, and this was more prominent in normal/underweight women who perceived themselves as overweight^8,13,14^.

Despite noted increases in the prevalence of obesity, the number of individuals who perceive themselves as overweight/obese has decreased, especially among overweight/obese people, in Western countries, suggesting a potential discrepancy between health professionals and lay persons in defining obesity. Moreover, increasing obesity and “normalized” overweight and obesity are proposed as additional reasons for this trend^6,15,16^. However, in Asian countries, where rapidly rising trends of obesity have been observed due to rapid urbanization followed by lifestyle changes including decreased physical activity and an increasingly energy-dense diet^17^, the proportion of individuals who perceive themselves as overweight is reportedly much higher than that of individuals who are actually overweight^18^, suggesting more distorted body weight perceptions. Another study also reported that proportions of women who perceive themselves as overweight and are trying to lose weight are higher in women residing in Asian countries, compared to Western countries, in all body mass index (BMI) deciles, especially women in lower BMI deciles^11^.

Previous studies of body weight perceptions and weight control behaviors have primarily been conducted in Western countries, focusing mostly on adolescents^8,9,12–14^. Studies in Asian countries are limited, especially those targeting general adult women. Korea’s urbanization rate is higher than other Asian countries^17^ and the effect of weight status, including both underweight and obesity, on subjective health status was higher than other Asian countries, which showed no association or association only with one of the two (underweight or obesity)^19^. Therefore, in this study, we aimed to investigate associations among self-perceived weight status, accuracy of weight perceptions based on BMI calculated from self-reported height and weight, and weight control behaviors, including both healthy and unhealthy behaviors, in a large, nationally representative sample from Korea.

## Methods

### Study design

We examined data from the 2016 Korean Study of Women’s Health Related Issues (K-Stori), a nationally representative cross-sectional survey undertaken from April 2016 to June 2016^20^. The goal of K-Stori was to investigate health awareness and needs according to the life cycles of women. Multistage stratified probability proportional to size systematic sampling was conducted. Trained interviewers contacted selected households and assessed the study eligibility of the residents. After obtaining informed consent from all eligible residents, surveys were conducted using a structured questionnaire. In addition, an online survey was conducted only among adolescent women. This study protocol was approved by the Institutional Review Board of the National Cancer Center, Korea (Approval no: NCC2016-0062). All procedures were performed in accordance with the Declaration of Helsinki 7^th^ version and informed consent was obtained from all participants. The details of K-Stori is described elsewhere^20^.

Of the 37,334 eligible people whom we contacted, 15,084 agreed to participate and completed the survey for a response rate of 40.4%. 6,178 of them had been excluded because they were less than 19 years of age, were pregnant, or were in the postpartum period. Finally, a total of 9,000 women were included initially for analysis.

### Patient and public involvement

Participants were not involved in the study design or conduct of the study. The results of the study were not disseminated among them.

### Body weight status and accuracy of weight perceptions

BMI was calculated based on self-reported height and weight. Weight status for the participants was categorized as underweight (<18.5 kg/m^2^), normal (≥18.5 kg/m^2^ and <23 kg/m^2^), overweight (≥23 kg/m^2^ and <25 kg/m^2^), and obese (≥25 kg/m^2^) according to obesity criteria for Asians^21^.

The participants were asked to describe their self-perceived body image as either very underweight, underweight, about right, overweight, or obese. We then categorized responses into four categories: underweight (combining very underweight and underweight), normal, overweight, or obese.

We then compared each participants’ weight status based on BMI group with their self-perceived body image. If the participant’s self-perceived body image corresponded with their weight status, they were classified into the accurately assessment group. If the participant’s self-perceived body image was below their weight status, they were classified into the underassessment group. Otherwise, they were classified into the overassessment group. For obese women, those who described their self-perceived body image as either overweight or obese were classified into the accurate assessment group.

### Weight control behaviors

Participants were asked to report how they had tried to control their weight during the previous 12 months as follows: lose weight, maintain weight, gain weight, or nothing. If they replied that they had ever tried to lose, maintain, or gain weight, they were further asked to describe all of the ways they had tried to control their weight among 10 options, including ‘exercising’, ‘fasting or skipping meals’, ‘dieting (eating less)’, ‘taking diet pills’, ‘taking over the counter diet pills’, ‘taking herbal medicine’, ‘taking dietary supplements’, ‘eating only one kind of food’, ‘undergoing a medical procedure’, and ‘others’. We classified these 10 options into six weight control behaviors as exercising; fasting or skipping meals; diet (eating less); taking diet pills, including ‘taking diet pills’, ‘taking over the counter diet pills’, ‘taking herbal medicine’, and ‘taking dietary supplements’; eating only one kind of food; and others.

### Statistical analysis

We limited the study sample to 8,584 normal weight, overweight, or obese women after excluding 416 underweight women. These underweight women were excluded because only 45 of them had tried to lose weight during the last 12 months, and this number was insufficient to obtain relevant adjusted results.

First, socio-demographic characteristics between normal weight, overweight, and obese women were compared using the chi-square test for contingency tables and one-way analysis of variance (ANOVA) for continuous variables. Then, the classifications of the participants according to weight status, perceived weight status, accuracy of weight perception, and weight control attempt were analyzed, especially the weight control behavior of women who tried to lose weight during the last 12 months.

Logistic regression was used to investigate whether weight status, perceived weight, and accuracy of weight perception were associated with trying to lose weight after adjusting for socio-demographic characteristics, including age as a continuous variable, residential area, education, marital status, household income, smoking and alcohol drinking status, and perceived health status. Among those who had ever tried to lose weight during the previous 12 months, the associations among weight status, perceived weight, accuracy of weight perception, and weight control behaviors were estimated using multivariable logistic regression analysis. In this analysis, five types of weight-control behaviors (exercising, fasting or skipping meals, eating less, taking diet pills, and eating only one kind of food) were considered and analyzed as dichotomous outcome variables (no vs. yes). In addition, subgroup analysis was conducted to highlight associations between accuracy of weight perceptions and trying to lose weight and weight control behaviors according to body weight status. Odds ratios (ORs) and 95% confidence intervals (95% CI) are presented as an index of association. Analyses were performed using R software (version 3.2.2).

### Ethical approval and informed consent

All patients gave written consent form. This study protocol was approved by the Institutional Review Board of the National Cancer Center, Korea (Approval no: NCC2016-0062).

## Results

Table 1 outlines the sociodemographic characteristics of the women aged 19–79 years according to their weight status (normal weight, overweight or obese). In comparison with women with normal weight, those who were overweight and obese belonged to the older age group, had lower education status, were less often single, had less household income, smoked less, drank less, and perceived their health status as unhealthy (P-value < 0.05). Discrepancies were discovered between actual weight status based on BMI and weight perceptions. 12.8% of normal weight women perceived that they were underweight, and 15.2% and 2.7% of them perceived that they were overweight and obese, respectively. 43.0% and 1.4% of overweight women perceived themselves as having the correct weight and being underweight; 2.4% of them perceived that they were obese. 17.0% and 0.4% of obese women perceived themselves as having the correct weight and being underweight, respectively. 22.8% of normal weight women had tried to lose weight during the last 12 months, which increased to 34.5% in overweight women and to 41.3% in obese women. However, about half of the overweight (47.8%) and obese women (46.0%) had not tried to control their weight during the previous 12 months. In addition, the sociodemographic characteristics of women classified according to their weight control attempt were presented in Appendix Table 1.

**Table 1:** Sociodemographic characteristics, self-perceptions of weight, and percentages of weight-control behaviors over the previous 12 months of the participants according to weight status.

Characteristics	Normal	Overweight	Obese	P-value
	(N = 4839)	(N = 2068)	(N = 1677)	
Age, mean (SD)	47.6 (17.6)	58.8 (14.2)	62.4 (13.3)	<0.001
Age group, N (%)				
19–39	1865 (38.5%)	226 (10.9%)	138 (8.2%)	<0.001
40–59	1536 (31.7%)	715 (34.6%)	413 (24.6%)	
60–69	685 (14.2%)	564 (27.3%)	488 (29.1%)	
70–79	753 (15.6%)	563 (27.2%)	638 (38.0%)	
Residential area, N (%)				
Urban	3859 (79.7%)	1624 (78.5%)	1351 (80.6%)	0.290
Rural	980 (20.3%)	444 (21.5%)	326 (19.4%)	
Education, N (%)				
≤Elementary school	717 (14.8%)	554 (26.8%)	612 (36.5%)	<0.001
Middle school	473 (9.8%)	377 (18.2%)	368 (21.9%)	
High school	1493 (30.9%)	760 (36.8%)	509 (30.4%)	
College or more	2156 (44.6%)	377 (18.2%)	188 (11.2%)	
Marital status, N (%)				
Single	1163 (24.0%)	113 (5.5%)	55 (3.3%)	<0.001
Cohabitant	3078 (63.6%)	1579 (76.4%)	1227 (73.2%)	
Divorced or widow	598 (12.4%)	376 (18.2%)	395 (23.6%)	
Household income, N (%)				
<2,000$/month	951 (19.7%)	647 (31.3%)	686 (40.9%)	<0.001
2,000–3,999$/month	1956 (40.4%)	866 (41.9%)	628 (37.4%)	
≥4,000$/month	1932 (39.9%)	555 (26.8%)	363 (21.6%)	
Smoking, N (%)				
Never	4562 (94.3%)	1985 (96.0%)	1594 (95.1%)	0.012
Ever	277 (5.7%)	83 (4.0%)	83 (4.9%)	
Drinking, N (%)				
Never	1058 (21.9%)	501 (24.2%)	433 (25.8%)	0.002
Ever	3781 (78.1%)	1567 (75.8%)	1244 (74.2%)	
Perceived health status, N (%)				
Healthy	2847 (58.8%)	923 (44.6%)	607 (36.2%)	<0.001
Normal	1423 (29.4%)	752 (36.4%)	662 (39.5%)	
Unhealthy	569 (11.8%)	393 (19.0%)	408 (24.3%)	
Perceived weight				
Underweight	620 (12.8%)	28 (1.4%)	6 (0.4%)	<0.001
Right weight	3352 (69.3%)	889 (43.0%)	285 (17.0%)	
Overweight	734 (15.2%)	1101 (53.2%)	1117 (66.6%)	
Obese	133 (2.7%)	50 (2.4%)	269 (16.0%)	
Accuracy of weight perception				
Underassessment	620 (12.8%)	917 (44.3%)	291 (17.4%)	<0.001
Accurate assessment	3352 (69.3%)	1101 (53.2%)	1386 (82.6%)	
Overassessment	867 (17.9%)	50 (2.4%)	—	
Attempts at weight control				
Trying to lose weight	1104 (22.8%)	714 (34.5%)	693 (41.3%)	<0.001
Trying to maintain weight	974 (20.1%)	349 (16.9%)	203 (12.1%)	
Trying to gain weight	74 (1.5%)	17 (0.8%)	9 (0.5%)	
Not trying to change or keep	2687 (55.5%)	988 (47.8%)	772 (46.0%)	

Dieting (less eating) was the most common weight control behavior to lose weight (about 70%), followed by exercise (about 50–60%) and fast/skipping meals (about 20–30%) among normal, overweight, and obese women (Table 2).

**Table 2:** Weight control behaviors to lose weight according to weight status.

	Trying to lose weight	Weight control behaviors among women who had tried to lose weight					
		Exercise	Fasting/meal skipping	Diet (ate less)	Diet pill	One kind of food	Others
Total	2511 (29.2%)	1313 (52.3%)	568 (22.6%)	1892 (75.3%)	404 (16.1%)	216 (8.6%)	12 (0.5%)
Normal (N = 4839)	1104 (22.8%)	544 (49.3%)	280 (25.4%)	816 (73.9%)	175 (15.9%)	121 (11.0%)	5 (0.5%)
Overweight (N = 2068)	714 (34.5%)	392 (54.9%)	142 (19.9%)	550 (77.0%)	107 (15.0%)	50 (7.0%)	2 (0.3%)
Obese (N = 1677)	693 (41.3%)	377 (54.4%)	146 (21.1%)	526 (76.0%)	122 (17.6%)	45 (6.5%)	5 (0.7%)

Perceived weight was more strongly associated with weight control attempts than actual weight status. Overweight and obese women showed increased odds of weight control attempts, compared with normal weight women (OR = 1.46 [95% CI = 1.27–1.68] and OR = 1.59 [95% CI = 1.36–1.87], Table 3). In women who perceived themselves as underweight, the odds for weight control attempts, compared to those who perceived themselves as right weight, was 0.34 (95% CI = 0.24–0.46). Meanwhile, those who perceived themselves as obese had tried to lose weight at an odds six times greater than for those with accurate weight perceptions (OR = 6.52, 95% CI = 5.21–8.17). Among women who tried to lose weight, obese women were more likely to have engaged in using diet pills (OR = 1.53, 95% CI = 1.10–2.12). Women who perceived themselves as obese were more likely to have engaged in exercise, fasting/meal skipping, and using diet pills. However, those who perceived themselves as underweight showed decreased odds of dieting (ate less) (OR = 0.47, 95% CI = 0.25–0.87), while those who perceived themselves as overweight showed increased odds of dieting (OR = 1.29, 95% CI = 1.03–1.62). According to accuracy of weight perceptions, women who under-assessed their weight were 58% less likely to have tried to lose weight during the last 12 month, while those who over-assessed their weight were twice more likely to lose weight, compared to women who accurately perceived their weight status. Women who under-perceived their weight were more likely to have engaged in fasting/meal skipping to reduce their weight (OR = 1.47, 95% CI = 1.07–1.99). Women who under-assessed their weight showed decreased odds for dieting (ate less) (OR = 0.57, 95% CI = 0.43–0.75).

**Table 3:** Adjusted odds ratios of weight lose behaviors according to weight status and perceived weight among the participants separately.

	Trying to lose weight	Weight control behaviors among women who had tried to lose weight				
		Exercise	Fasting/meal skipping	Dieting (ate less)	Diet pill	One kind of food
Weight status^a^						
Normal	1 (Ref)	1 (Ref)	1 (Ref)	1 (Ref)	1 (Ref)	1 (Ref)
Overweight	1.46 (1.27–1.68)	1.12 (0.90–1.40)	1.12 (0.86–1.46)	1.03 (0.80–1.33)	1.17 (0.86–1.58)	0.75 (0.50–1.11)
Obese	1.59 (1.36–1.87)	0.99 (0.78–1.26)	1.29 (0.96–1.72)	0.96 (0.73–1.26)	1.53 (1.10–2.12)	0.80 (0.51–1.23)
Perceived weight^b^						
Underweight	0.34 (0.24–0.46)	1.72 (0.92–3.28)	1.56 (0.80–2.93)	0.47 (0.25–0.87)	1.42 (0.59–3.04)	1.48 (0.58–3.31)
Right weight	1 (Ref)	1 (Ref)	1 (Ref)	1 (Ref)	1 (Ref)	1 (Ref)
Overweight	3.49 (3.09–3.96)	0.93 (0.77–1.14)	0.91 (0.71–1.15)	1.29 (1.03–1.62)	1.55 (1.17–2.08)	1.26 (0.89–1.80)
Obese	6.52 (5.21–8.17)	1.36 (1.00–1.85)	1.50 (1.05–2.13)	1.13 (0.80–1.61)	3.12 (2.08–4.66)	1.07 (0.58–1.87)

^a^Adjusted for age, residential area, marital status, education, household income, smoking, drinking, perceived health status, and perceived weight.

^b^Adjusted for age, residential area, marital status, education, household income, smoking, drinking, perceived health status, and weight status.

The associations between weight misperception and weight control behaviors according to weight status are presented in Table 4. Normal weight women who over-assessed their weight had tried to lose weight at an odds about four times higher than that in normal weight women; no significant difference was observed in overweight women who over-assessed their weight. Among normal weight women who had tried to lose weight, those who perceived themselves as overweight were more likely to report using diet pills (OR = 1.62, 95% CI = 1.15–2.29), while their odds of exercise and dieting (ate less) were about 1, compared to normal weight women with correct weight perceptions. Normal weight women who under-assessed their weight, however, were less likely to have dieted (ate less). Among overweight women, those with weight misperceptions showed an increased odds of engaging in fasting/meal skipping during the last 12 months (OR = 1.61 [95% CI = 1.03–2.49] in under-assessing women and OR = 5.72 [95% CI = 2.45–13.56] in over-assessing women). Overweight women who under-assessed their weight showed a reduced odds of having dieted (ate less), and overweight women who over-assessed their weight showed a significantly higher odds of having taken diet pills (OR = 3.16 (95% CI = 1.15–8.23), compared with those who accurately assessed their weight. The crude association of weight status and weight perception or misperception with weight control attempts and weight control behaviors is shown in Appendix Tables 2 and 3.

**Table 4:** Adjusted odds ratios of weight lose behaviors according to the combination of weight status and perceived weight among the participants.

	Trying to lose weight	Weight control behaviors among women who had tried to lose weight				
		Exercise	Fasting/meal skipping	Diet (ate less)	Diet pill	One kind of food
Normal						
Underassessment	0.32 (0.23–0.44)	1.71 (0.88–3.40)	1.66 (0.81–3.27)	0.42 (0.22–0.81)	1.38 (0.54–3.10)	1.30 (0.46–3.10)
Accurate assessment	1 (Ref)	1 (Ref)	1 (Ref)	1 (Ref)	1 (Ref)	1 (Ref)
Overassessment	4.34 (3.67–5.13)	0.99 (0.76–1.28)	1.21 (0.90–1.63)	1.01 (0.75–1.35)	1.62 (1.15–2.29)	1.12 (0.74–1.69)
Overweight						
Underassessment	0.31 (0.25–0.39)	1.05 (0.74–1.51)	1.61 (1.03–2.49)	0.52 (0.35–0.78)	0.62 (0.33–1.09)	0.51 (0.20–1.12)
Accurate assessment	1 (Ref)	1 (Ref)	1 (Ref)	1 (Ref)	1 (Ref)	1 (Ref)
Overassessment	1.58 (0.86–2.95)	1.15 (0.52–2.61)	5.72 (2.45–13.56)	0.55 (0.23–1.47)	3.16 (1.15–8.23)	0.72 (0.11–2.78)
Obese						
Underassessment	0.27 (0.19–0.38)	1.47 (0.80–2.76)	1.20 (0.57–2.36)	0.71 (0.38–1.38)	0.42 (0.13–1.07)	0.93 (0.21–1.83)
Accurate assessment	1 (Ref)	1 (Ref)	1 (Ref)	1 (Ref)	1 (Ref)	1 (Ref)

Adjusted for age, residential area, marital status, education, household income, smoking, drinking, and perceived health status.

## Discussion

This study outlined weight control behaviors in Korean women according to weight perceptions. We observed that a large number of women did not accurately assess their BMI category. Herein, 12.8%, 44.3%, and 17.4% of normal weight, overweight, and obese women under-assessed their weight, while 17.9% of normal weight women over-assessed their weight. About half of the overweight and obese women had not tried to control their weight, and weight perceptions were strongly associated with having tried to lose weight. Exercise and dieting (ate less), which are considered healthy weight control behaviors^8^, were the most commonly applied weight control behaviors in women who had tried to lose weight. However, misperception of weight status was related to more unhealthy weight control behaviors and fewer healthy weight control behaviors: Women who under-assessed their weight showed less of a tendency to engage in dieting and a greater tendency to fast or skip meals. Overweight women who over-assessed their weight were more likely to have engaged in fasting or skipping meals and using diet pills, compared to women with normal weight or accurate self-weight assessment.

An association between inaccurate weight perception and unhealthy weight control behaviors has been reported in several previous studies. However, details on the association vary between studies. In a study targeting women aged 18 to 25 years, normal weight women who perceived themselves as overweight participated in both healthy weight control behaviors, including dieting, and unhealthy behaviors, such as skipping meals, and using diet pills or diuretics more. Meanwhile, overweight women who under-assessed their weight participated in these healthy and unhealthy weight control behaviors less^8^. These results were consistent with findings in high school students, wherein normal weight students with overweight perceptions were more likely to rely on healthy weight control behaviors, such as exercise and dieting, and unhealthy behaviors, such as fasting, using diet pills, vomiting, or using laxatives^14^. In studies focusing only on unhealthy weight control behaviors, underweight and normal weight girls who over-assessed their weight engaged more often in unhealthy weight management practices^13,22,23^. Overweight girls who underestimated their weight have been shown to be less engaged in unhealthy weight control behaviors^13,24^. In general, underestimation of weight has been found to be associated with fewer unhealthy behaviors, with overestimation being associated with more unhealthy behaviors^18^.

In our results, however, all directions of weight misperception were related to unhealthy weight control behaviors. Normal weight women who over-assessed their weight had point estimates of ORs close to 1 for exercise and diet, suggesting that they are less likely to adopt healthy weight control behaviors and more likely to rely on diet pills than those who accurately assessed their weight. In addition, overweight women who under-assessed or over-assessed their weight status reported more unhealthy weight control behaviors and less healthy weight control behaviors. Several previous studies focusing on weight perceptions and weight control behaviors have indicated that both self-perceptions of being underweight and overweight are associated with unhealthy weight loss behaviors^25,26^. These results could support our findings that both under- and over-assessment of weight are associated with unhealthy weight loss behaviors in women, especially in overweight women. In obese women, although those who under-assessed their weight status reported less engagement in weight control by about 70%, their methods for achieving weight control were not significantly different from obese women with accurate weight assessment. When we classified obese women whose self-perceived body image was overweight as under assessed, there were no significant differences between the underassessment and accurate assessment groups in regards to weight control behaviors. To the best of our knowledge, this is the first study to report that both over-assessment and under-assessment of weight are associated with increased reliance on unhealthy weight control behaviors and decreased reliance on healthy weight control behaviors.

As another important finding from our study, unhealthy weight control behaviors were identified in obese women. While point estimates of ORs for exercise and diet were close to 1, the OR of diet pill use was significantly higher than that for normal weight women. These results are consistent with previous findings^18,25,27^. Although pharmacotherapy combined with intensive lifestyle modification is recommended for obese women (BMI ≥ 30 kg/m^2^) or women with BMI ≥ 27 kg/m^2^ and concurrent co-morbidities, only 12 women reported BMI ≥ 30 kg/m^2^ and none of those who took a diet pill conducted lifestyle modification, including both exercise and dietary measures.

Several limitations to the present study should be mentioned. First, BMI was calculated by self-reported weight and height. While self-reported weight and height are highly correlated with measured values^28^, in general, self-reported weight is underreported, especially in overweight and obese women, and height is over-reported, leading to misclassification of BMI categories^29,30^. Thus, there could be a possibility that the proportions of the overweight or obese women would be underestimated in this study, followed by greater underassessment of weight perceptions. When we compared the prevalence of overweight/obesity in this study with results from the Korean National Health and Nutrition Examination Survey, in which trained medical staff measured height and weight^31,32^, the prevalence herein was lower. However, the overall proportion of weight underassessment in this study (21.3%) was comparable to that in previous studies conducted for Korean adult women using Korean National Health and Nutrition Examination Survey data (about 25%)^33,34^. Thus, misperception of weight, especially underassessment would be more serious. However, several previous studies investigating the association between accurate weight perceptions and weight management patterns also used BMI categories from self-reported height and weight^13,14,18^, showing that measuring accuracy of weight perceptions based on BMI calculation from self-reported height and weight may be applicable. In addition, as a variable that reflects both real body weight and personal desires or perceptions^35^, and also as a variable that is more directly associated with perceived weight status^36^, BMI calculated from self-reported height and weight may be of merit despite possible misclassification. Further research that links actual BMI from objective measurements, BMI from self-reported height and weight, perceived weight status, body dissatisfaction, intended weight control, and actual weight control behavior would be beneficial.

Other limitations are as follows: The accuracy of weight perception was crudely classified as underassessment, accurate assessment, and overassessment. Thus, we could not reflect degrees of underassessment and overassessment; for example, both women who were near the overweight cutoff or who far exceeded the cutoff were classified in the same category. In addition, the time point of the assessment of BMI and body weight perceptions (at the time of survey) was different from that of weight control behaviors (during the past 12 months). Thus, drawing a causal relationship between accuracy of weight perception and weight control behaviors would be difficult or even reversed, due to the cross-sectional design of the study. However, only 630 out of 8,584 study participants reported that they had lost 3 kg or more weight compared to one year previously. Thus, we did not consider reverse causation between weight perception and weight control attempts to be a serious issue. K-Stori is a nationally representative study in which the weights of individuals were determined considering the sampling ratio and response rates. However, in this study, we used a subset of K-Stori subjects and focused on association between variables, rather than representativeness. Therefore, the results may include a selection bias due to a low response rate (40.4%), and do not represent the entire Korean female population. BMI cannot distinguish lean mass and fat mass, and thus, women with dense muscle mass could be misclassified as overweight. Nevertheless, studies have shown a strong correlation between BMI and body fat percentages^37^.

## Supplementary information

Supplementary tables

## Data Availability

Data is available on request to the Center for Disease Control and Prevention, Korea.

## References

[1] Ng M (2014). Global, regional, and national prevalence of overweight and obesity in children and adults during 1980–2013: a systematic analysis for the Global Burden of Disease Study 2013. Lancet.

[2] Haslam DW, James WP (2005). Obesity. Lancet.

[3] Wing RR (2011). Benefits of modest weight loss in improving cardiovascular risk factors in overweight and obese individuals with type 2 diabetes. Diabetes Care.

[4] Sumithran P, Proietto J (2016). Maintaining Weight Loss: an Ongoing Challenge. Curr Obes Rep.

[5] Gupta H (2014). Barriers to and Facilitators of Long Term Weight Loss Maintenance in Adult UK People: A Thematic Analysis. Int J Prev Med.

[6] Johnson F, Cooke L, Croker H, Wardle J (2008). Changing perceptions of weight in Great Britain: comparison of two population surveys. BMJ.

[7] Monteagudo C, Dijkstra SC, Visser M (2015). Self- Perception of Body Weight Status in Older Dutch Adults. J Nutr Health Aging.

[8] Rahman M, Berenson AB (2010). Self-perception of weight and its association with weight-related behaviors in young, reproductive-aged women. Obstet Gynecol.

[9] Alwan H, Viswanathan B, Paccaud F, Bovet P (2011). Is Accurate Perception of Body Image Associated with Appropriate Weight-Control Behavior among Adolescents of the Seychelles. J Obes.

[10] Lemon SC, Rosal MC, Zapka J, Borg A, Andersen V (2009). Contributions of weight perceptions to weight loss attempts: differences by body mass index and gender. Body Image.

[11] Wardle J, Haase AM, Steptoe A (2006). Body image and weight control in young adults: international comparisons in university students from 22 countries. Int J Obes (Lond).

[12] Duncan DT (2011). Does perception equal reality? Weight misperception in relation to weight-related attitudes and behaviors among overweight and obese US adults. Int J Behav Nutr Phys Act.

[13] Ibrahim C, El-Kamary SS, Bailey J, George DM (2014). Inaccurate weight perception is associated with extreme weight-management practices in U.S. high school students. J Pediatr Gastroenterol Nutr.

[14] Ursoniu S, Putnoky S, Vlaicu B (2011). Body weight perception among high school students and its influence on weight management behaviors in normal weight students: a cross-sectional study. Wien Klin Wochenschr.

[15] Burke MA, Heiland FW, Nadler CM (2010). From “overweight” to “about right”: evidence of a generational shift in body weight norms. Obesity (Silver Spring).

[16] Johnson F, Beeken RJ, Croker H, Wardle J (2014). Do weight perceptions among obese adults in Great Britain match clinical definitions? Analysis of cross-sectional surveys from 2007 and 2012. BMJ Open.

[17] Ramachandran A, Chamukuttan S, Shetty SA, Arun N, Susairaj P (2012). Obesity in Asia–is it different from rest of the world. Diabetes Metab Res Rev.

[18] Kim DS, Kim HS, Cho Y, Cho SI (2008). The effects of actual and perceived body weight on unhealthy weight control behaviors and depressed mood among adult women in Seoul, Korea. J Prev Med Public Health.

[19] Noh JW (2017). Body mass index and self-rated health in East Asian countries: Comparison among South Korea, China, Japan, and Taiwan. PLoS One.

[20] Cho HN (2017). The Korean Study of Women’s Health-Related Issues (K-Stori): Rationale and Study Design. BMC public health.

[21] Committee, S. The Asia-Pacific perspective: Redefining obesity and its treatment. *Melbourne: International Diabetes Institute*, 11–12 (2000).

[22] Talamayan KS, Springer AE, Kelder SH, Gorospe EC, Joye KA (2006). Prevalence of overweight misperception and weight control behaviors among normal weight adolescents in the United States. ScientificWorldJournal.

[23] Lee KM, Seo MS, Shim JY, Lee YJ (2015). Body weight status misperception and its association with weight control behaviours, depressive mood and psychological distress in nulliparous normal-weight young women. Ann Hum Biol.

[24] Sonneville KR, Thurston IB, Milliren CE, Gooding HC, Richmond TK (2016). Weight misperception among young adults with overweight/obesity associated with disordered eating behaviors. Int J Eat Disord.

[25] Haley CC, Hedberg K, Leman RF (2010). Disordered eating and unhealthy weight loss practices: which adolescents are at highest risk?. J Adolesc Health.

[26] Gonsalves D, Hawk H, Goodenow C (2014). Unhealthy weight control behaviors and related risk factors in Massachusetts middle and high school students. Matern Child Health J.

[27] Boutelle K, Neumark-Sztainer D, Story M, Resnick M (2002). Weight control behaviors among obese, overweight, and nonoverweight adolescents. J Pediatr Psychol.

[28] Spencer EA, Appleby PN, Davey GK, Key TJ (2002). Validity of self-reported height and weight in 4808 EPIC-Oxford participants. Public Health Nutr.

[29] Tang W, Aggarwal A, Moudon AV, Drewnowski A (2016). Self-reported and measured weights and heights among adults in Seattle and King County. BMC Obes.

[30] Skeie G, Mode N, Henningsen M, Borch KB (2015). Validity of self-reported body mass index among middle-aged participants in the Norwegian Women and Cancer study. Clin Epidemiol.

[31] Kang HT (2014). Trends in prevalence of overweight and obesity in Korean adults, 1998–2009: the Korean National Health and Nutrition Examination Survey. J Epidemiol.

[32] Korea National Health & Nutrition Examination Survey Web site. Available at, https://knhanes.cdc.go.kr/knhanes/eng/index.do.

[33] Joh HK, Oh J, Lee HJ, Kawachi I (2013). Gender and socioeconomic status in relation to weight perception and weight control behavior in Korean adults. Obes Facts.

[34] Hwang JH, Ryu DH, Park SW (2015). Interaction Effect between Weight Perception and Comorbidities on Weight Control Behavior in Overweight and Obese Adults: Is There a Sex Difference?. J Korean Med Sci.

[35] Villanueva EV (2001). The validity of self-reported weight in US adults: a population based cross-sectional study. BMC Public Health.

[36] Wang Y, Liang H, Chen X (2009). Measured body mass index, body weight perception, dissatisfaction and control practices in urban, low-income African American adolescents. BMC Public Health.

[37] Flegal KM (2009). Comparisons of percentage body fat, body mass index, waist circumference, and waist-stature ratio in adults. Am J Clin Nutr.

