# Supplementary material for: Weight control behaviors according to body weight status and accuracy of weight perceptions among Korean women: a nationwide population-based survey
Source: Sci Rep. 2019 Jun 24;9:9127. doi: 10.1038/s41598-019-45596-z (PMC6591250; doi:10.1038/s41598-019-45596-z)
Supplement: Supplementary file 1 — Supplementary tables [file 41598_2019_45596_MOESM1_ESM.pdf]

Weight control behaviors according to body weight status and accuracy of weight perceptions among Korean women: a nationwide population-based survey

Boyoung Park[1] <hayejine@hanmail.net>

Ha Na Cho[2] <hana@ncc.re.kr>

Eunji Choi[2] <eunji.choi@ncc.re.kr>

Da Hea Seo[3] <DAHEA@yuhs.ac>

Nam-Soon Kim[4] <artemine@kihasa.re.kr>

Eunja Park[4] <ejpark@kihasa.re.kr>

Sue Kim[5] <SUEKIM@yuhs.ac>

Yeong-Ran Park[6] <yeongranpark@daum.net>

Kui Son Choi[2*] <kschoi@ncc.re.kr>

Yumie Rhee[7*] <yumie@yuhs.ac>

[1]Department of Medicine, Hanyang University College of Medicine

[2]Department of Cancer Control and Population Health, Graduate School of Cancer Science and Policy, National Cancer Center

[3]Department of Endocrinology and Metabolism, School of Medicine, Inha University

[4]Health Care Policy Research Department, Korea Institute for Health and Social Affairs

[5]College of Nursing, Yonsei University

[6]Department of Silver Industry, Kangnam University

[7]Department of Internal Medicine, Endocrine Research Institute, Severance Hospital, Yonsei University College of Medicine

*Corresponding author: KS Choi and Y Rhee contributed equally

Appendix Table 1. Distribution of covariates according to weight status according to weight lose behaviors (trying to lose weight)

Characteristics	Not trying to lose weight (N=6073)	Trying to lose weight (N=2511)	P-value
Age, mean (SD)	54.5 ± 17.5	49.9 ± 16.6	<0.001
Age group, N (%)			
19 -39	1477 (24.3%)	752 (29.9%)	<0.001
40-59	1731 (28.5%)	933 (37.2%)	
60-69	1274 (21.0%)	463 (18.4%)	
70-79	1591 (26.2%)	363 (14.5%)	
Residential area, N (%)			
Urban	4843 (79.7%)	1991 (79.3%)	0.655
Rural	1230 (20.3%)	520 (20.7%)	
Education, N (%)			
≤Elementary school	1555 (25.6%)	328 (13.1%)	<0.001
Middle school	905 (14.9%)	313 (12.5%)	
High school	1834 (30.2%)	928 (37.0%)	
College or more	1779 (29.3%)	942 (37.5%)	
Marital status, N (%)			
Single	877 (14.4%)	454 (18.1%)	<0.001
Cohabitant	4100 (67.5%)	1784 (71.0%)	
Divorced or widow	1096 (18.0%)	273 (10.9%)	
Household income, N (%)			
<2,000$/month	1806 (29.7%)	478 (19.0%)	<0.001
2,000-3,999$/month	2384 (39.3%)	1066 (42.5%)	
≥4,000$/month	1883 (31.0%)	967 (38.5%)	
Smoking, N (%)			
Never	5799 (95.5%)	2342 (93.3%)	0.012
Ever	274 (4.5%)	169 (6.7%)	
Drinking, N (%)			
Never	1581 (26.0%)	411 (16.4%)	0.002
Ever	4492 (74.0%)	2100 (83.6%)	
Perceived health status, N (%)			
Healthy	3060 (50.4%)	1317 (52.4%)	<0.001
Normal	1936 (31.9%)	901 (35.9%)	
Unhealthy	1077 (17.7%)	293 (11.7%)	

Appendix Table 2. Crude odds ratios of weight lose behaviors (trying to lose weight) according to weight status and weight perceptions among the participants

	Trying to lose weight	Weight control behaviors among women who had tried to lose weight				
		Exercise	Fasting/meal skipping	Dieting (ate less)	Diet pill	One kind of food
Weight status[1]						
Normal	1 (Ref)	1 (Ref)	1 (Ref)	1 (Ref)	1 (Ref)	1 (Ref)
Overweight	1.78 (1.59-2.00)	1.25 (1.04-1.51)	0.73 (0.58-0.92)	1.18 (0.95-1.48)	0.94 (0.72-1.21)	0.61 (0.43-0.86)
Obese	2.38 (2.12-2.68)	1.23 (1.02-1.49)	0.79 (0.62-0.98)	1.11 (0.89-1.39)	1.13 (0.88-1.46)	0.56 (0.39-0.80)
Perceived weight [2]						
Underweight	0.31 (0.23-0.42)	1.63 (0.89-3.07)	1.70 (0.89-3.16)	0.47 (0.25-0.87)	1.50 (0.64-3.15)	1.84 (0.73-4.01)
Right weight	1 (Ref)	1 (Ref)	1 (Ref)	1 (Ref)	1 (Ref)	1 (Ref)
Overweight	3.47 (3.13-3.86)	1.05 (0.89-1.25)	0.77 (0.63-0.95)	1.29 (1.03-1.62)	1.37 (1.08-1.77)	0.91 (0.68-1.24)
Obese	6.04 (4.94-7.39)	1.52 (1.15-2.01)	1.19 (0.87-1.62)	1.13 (0.80-1.61)	2.34 (1.66-3.29)	0.72 (0.41-1.20)

Appendix Table 3. Crude odds ratios of weight lose behaviors (trying to lose weight) according to weight status and accuracy of weight perceptions among the participants

	Trying to lose weight	Weight control behaviors among women who had tried to lose weight				
		Exercise	Fasting/meal skipping	Diet (ate less)	Diet pill	One kind of food
Normal						
Underassessment	0.29 (0.20-0.40)	1.64 (0.88-3.40)	1.64 (0.82-3.18)	0.41 (0.22-0.80)	1.33 (0.53-2.94)	1.46 (0.54-3.36)
Accurate assessment	1 (Ref)	1 (Ref)	1 (Ref)	1 (Ref)	1 (Ref)	1 (Ref)
Overassessment	3.85 (3.29-4.51)	1.11 (0.87-1.42)	1.04 (0.78-1.38)	0.99 (0.75-1.32)	1.47 (1.06-2.05)	1.00 (0.67-1.48)
Overweight						
Underassessment	0.27 (0.22-0.33)	1.09 (0.77-1.54)	1.51 (0.98-2.28)	0.52 (0.35-0.76)	0.52 (0.29-0.90)	0.48 (0.19-1.03)
Accurate assessment	1 (Ref)	1 (Ref)	1 (Ref)	1 (Ref)	1 (Ref)	1 (Ref)
Overassessment	1.46 (0.83-2.62)	1.12 (0.52-2.47)	4.89 (2.23-10.70)	0.74 (0.32-1.91)	2.07 (0.83-4.69)	0.88 (0.14-3.10)
Obese						
Underassessment	0.24 (0.17-0.33)	1.54 (0.85-2.84)	1.35 (0.67-2.54)	0.65 (0.36-1.24)	0.50 (0.17-1.17)	0.91 (0.22-2.63)
Accurate assessment	1 (Ref)	1 (Ref)	1 (Ref)	1 (Ref)	1 (Ref)	1 (Ref)
